# Mental health of nursing professionals: Internet-based interventions

**DOI:** 10.47626/1679-4435-2022-801

**Published:** 2023-08-08

**Authors:** Regina de Souza Moreira, Magno Conceição das Merces, Alex Almeida e Almeida, Thais Regis Aranha Rossi, Helena Fraga Maia, Argemiro D’Oliveira-Júnior

**Affiliations:** 1 Programa de Pós-Graduação em Ciências da Saúde, Faculdade de Medicina da Bahia, Universidade Federal da Bahia (UFBA), Salvador, BA, Brazil; 2 Departamento de Ciências da Vida, Universidade do Estado da Bahia (UNEB), Salvador, BA, Brazil; 3 Clínica Transcender, Salvador, BA, Brazil

**Keywords:** mental health, nurses, review, Internet-based intervention, saúde mental, enfermeiras e enfermeiros, revisão, intervenção baseada em internet

## Abstract

This study aimed to analyze published evidence about how the Internet is used in mental health interventions for nurses. This integrative literature review searched MEDLINE (PubMed), Literatura Latino-Americana e do Caribe em Ciências da Saúde (LILACS), Base de Dados em Enfermagem (BDENF), and the Web of Science. Data was collected in July 2020. Six articles addressed the research question – “What Internet-based mental health interventions exist for nurses?” –, of which five were randomized clinical trials (2 protocols and 3 completed trials) and 1 was a cohort study. Identified intervention programs consisted of 4 interactive webpages and 2 smartphone apps, based on cognitive behavioral and holistic approaches. The intervention programs were effective in reducing stress, anxiety, and depression among nurses; however, there was a lack of scientific evidence on the subject and a research gap regarding other approaches.

## Abstract (pt)

Este estudo objetivou analisar as evidências científicas encontradas na literatura sobre como se configuram as intervenções na saúde mental dos enfermeiros baseadas na utilização da internet. Tratou-se de revisão integrativa da literatura, conduzida nas bases de dados MEDLINE (PubMed), Literatura Latino-Americana e do Caribe em Ciências da Saúde (LILACS), Base de Dados em Enfermagem (BDENF) e Web of Science. A coleta de dados foi realizada em julho de 2020. Foram identificados seis artigos, por meio dos quais foi possível responder à pergunta de investigação: “Quais são as intervenções na saúde mental de enfermeiros baseadas na utilização da internet?”. Identificaram-se cinco ensaios clínicos randomizados (dois protocolos e três ensaios realizados) e um estudo de coorte. Os programas identificados consistiram em quatro páginas interativas virtuais e dois aplicativos para *smartphones*, com abordagens cognitivo-comportamentais e holísticas. Os programas de intervenção foram eficazes na redução do estresse, ansiedade e depressão em enfermeiros; contudo, notou-se escassez de evidências científicas sobre a temática, além de uma lacuna atinente a outras vertentes de abordagem.

## INTRODUCTION

Caring for human beings is the essence of nursing. However, the conditions and characteristics of this profession can lead to physical and mental illness.^1^ This is evident considering context in which nurses perform their work, ie, high stress levels, exhausting working hours, scant resources, exposure to a variety of feelings and emotions, and other factors that can negatively affect their mental health.^1,2^

In the current context of the COVID-19 pandemic, this scenario becomes even more complex, given that frontline healthcare professionals are among the most affected groups due the risk of contagion and emotional pain and vulnerability to anxiety, fear, anguish, lowering of mood, and emotional exhaustion. Of note, this situation is exacerbated by missing or insufficient personal protective equipment, high stress levels due changing work conditions, and tension due to the many COVID-19 cases and deaths. These professionals are often affected by fear of becoming infected or infecting family members, loneliness due to social distancing, and the loss of co-workers and/or family members.^3,4^

Research has shown that nursing professionals are affected by wear on their mental health.^2,5,6,7,8,9,10^ A study on nurses who work with COVID-19 patients in Wuhan, China found high rates of sleep disorders (60%) and symptoms of depression (46%) and anxiety (40%).^11^ Likewise, a 2020 study on nurses at a reference hospital for COVID-19 in Paraná, Brazil found high levels of anxiety (48.9%) and depression (25%).^12^ However, few studies have emphasized intervention strategies to mitigate such issues.

Considering that health condition and quality of life affect work satisfaction and productivity, in addition to patient care, interventions to reduce mental illness among nurses are extremely important.^1^ Thus, due to the emerging need for mental health care among nurses during the COVID-19 pandemic, the Brazilian Federal Nursing Commission established the National Commission for Mental Health in Nursing to provide online assistance via live chat with professionals with specialist, masters, or doctoral degrees in mental health.^3^

A meta-analysis found that Internet-based interventions reduced occupational stress in general workers^13^ and improved depressive symptoms.^14^ It goes without saying that the Internet, which has helped universalize information,^15^ has assumed a prominent place in modern society; its interactivity, ease of use, and speed are strong attractions. During the pandemic, it grew in importance due to the need for social distancing to control virus transmission. Thus, the virtual environment has become an essential resource for social interaction, as well as for the continuity of activities in widely different areas of society, including health care.

Given the above and based on the premise that nursing professionals need better mental health care, in addition to the importance of technological resources and the daily online activity of the general population, this study aimed to review virtual Internet-based health intervention strategies for nurses.

## OBJECTIVES

This review’s purpose was to analyze scientific evidence about Internet-based mental health interventions for nurses.

## METHODS

### ETHICAL ASPECTS

Since this was a literature review, research ethics committee approval was waived. However, all ethical precepts related to the analyzed studies were respected.

### STUDY TYPE AND METHODOLOGICAL PROCEDURES

This integrative literature review did the following: (i) identified the subject and elaborated the following research question “What Internet-based mental health interventions exist for nurses?”; (ii) established inclusion and exclusion criteria; (iii) searched for articles in the selected databases; (iv) identified a sample of studies for analysis; (v) categorized the selected studies, analyzing and interpreting the results; and (vi) presented an overview of the review.^16^

### DATA COLLECTION AND ORGANIZATION

Data was collected between July and October 2020 from MEDLINE (PubMed), Literatura Latino-Americana e do Caribe em Ciências da Saúde (LILACS), Base de Dados de Enfermagem (BDENF), and the Web of Science. To search for articles, the following Descritores em Ciências da Saúde search terms were selected: “intervenção baseada em Internet”/”Internet-based intervention”, “enfermagem”/”nurse”, “saúde mental”/”metal health”, “depressão”/“depression”, “burnout”, “ansiedade”/“anxiety” and “estresse”/“stress”. These terms were associated with the Boolean operators *AND* and *OR*, according to the search strategies outlined in the Chart 1.

**Chart 1:** Article search strategies

Data base	DeCS combinations
MEDLINE (66 articles)	(Internet based intervention) AND ((mental health) OR (depression) OR (burnout) OR (stress) OR (anxiety)) AND (nurse*)
Web of Science (95 articles)	ALL = ((Internet based intervention OR ehealth) AND mental health AND nurse)
LILACS/BDENF (72 articles)	(Internet based intervention) AND (mental health) AND (nurse)

BDENF = Bases de Dados de Enfermagem; DeCS = Descritores em Ciências da Saúde; LILACS = Literatura Latino-Americana e do Caribe em Ciências da Saúde.

The inclusion criteria were defined as follows: articles published in Brazilian or international journals in English, Portuguese, or Spanish that addressed online mental health interventions for nursing professionals and whose full text was available. Articles whose scientific evidence level classification ranged from 1 to 3 according to the Preferred Reporting Items for Systematic Reviews and Meta-Analyses (PRISMA) were also included. No starting or ending publication dates were set. Documents, editorials, letters, theses, dissertations, monographs, manuals, and conference abstracts were excluded, as well as duplicate articles in more than one database or articles that did not answer the research question.

### DATA ANALYSIS

**Figure 1:**
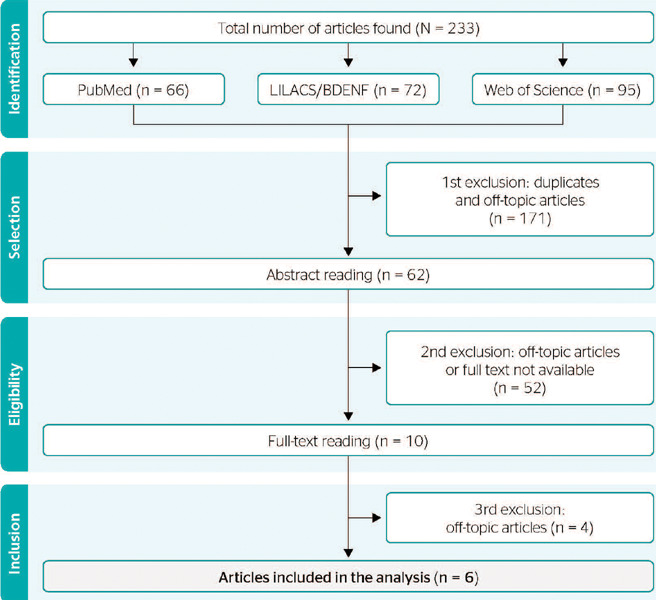
Diagram of the article selection process. BDENF = Bases de Dados de Enfermagem; LILACS = Literatura Latino-Americana e do Caribe; PubMed = Publisher MEDLINE.

Of the total number of articles found, 40.8% were identified in the Web of Science, 30.9% in LILACS/BDENF, and 28.3% in PubMed. Articles that were not available electronically or that were published in other languages were excluded. After reading the titles of the articles, those whose theme did not align with this review or whose study population did not involve nursing professionals were excluded. Articles whose population was of informal caregivers, parents, adolescents, children, the chronically ill, cancer patients, etc. were excluded. Duplicate articles were then excluded. Finally, a new content relevance analysis was carried out. After a detailed reading of the full texts, 1 article was excluded, an observational study analyzing nurses’ adherence to lifestyle change apps, since mental health aspects were not analyzed. Figure 1 details the selection process.

The following criteria were used to categorize the studies: year/country, title, author, journal, study design/level of evidence (LE), and population/scenario (Table 1). The research question was answered through analysis of the identified articles, ie, which intervention programs are described in the literature, their approach, and the instruments they used to assess mental health.

**Table 1:** Summary of included studies according to year/country, title, author, journal, study design/LE, and population/scenario.

Year/country	Title	Author(s)	Journal	Study design/LE	Population/scenario
2016/USA (VA & NY)	Reducing nurses’ stress: a randomized controlled trial of a web-based stress management program for nurses	Hersch et al.^17^	Applied Nursing Research	Randomized clinical trial/2	Nurses/large hospital
2019/Vietnam	Effects of two types of smartphone-based stress management programs on depressive and anxiety symptoms among hospital nurses in Vietnam: a protocol for three-arm randomized controlled trial	Imamura et al.^18^	BMJ Open	Randomized clinical trial protocol/2	Nurses/large hospital
2019/Japan	Effects of an Internet-based cognitive behavioral therapy intervention on improving depressive symptoms and work- related outcomes among nurses in Japan: a protocol for a randomized controlled trial	Kuribayashi et al.^19^	BMC Psychiatry	Randomized clinical trial protocol/2	Nurses/hospitals
2018/ USA (VA)	Evaluation of a web-based holistic stress reduction pilot program among nurse-midwives	Wright^20^	Journal of Holistic Nursing	Cohort study/4	Nurse midwives/hospitals, practices, birth centers, home births, and higher education settings
2020/Holland	Help in hand after traumatic events: a randomized controlled trial in health care professionals on the eficacy, usability, and user satisfaction of a self-help app to reduce trauma-related symptoms	Van der Meer et al.^21^	European Journal of Psychotraumatology	Randomized clinical trial/2	Nurses and other healthcare workers/hospitals and ambulance services
2018/Germany	Promoting the self-regulation of stress in health care providers: an Internet-based intervention	Gollwitzer et al.^22^	Frontiers in Psychology	Randomized clinical trial/2	Nurses/hospitals, nursing homes, residential care, psychiatric institutions, rehabilitation centers, etc.

BMJ = British Medical Journal; BMC = BioMed Central; LE = level of evidence.

## RESULTS

Six studies published in 2016, 2018, 2019, and 2020 were identified (2 each in 2018 and 2019). The studies were from Vietnam, the United States,

Japan, Germany, and Holland (ie, none from Brazil). The articles were published in the *British Medical Journal Open, BioMed Central Psychiatry*, *Applied Nursing Research*, the *Journal of Holistic Nursing*, *Frontiers in Psychology*, and the *European Journal of Psychotraumatology*. Regarding the type of research, most had an intervention study design: 2 were randomized clinical trials (RCT) protocols (LE 2), 3 were completed RCTs (LE 2), and 1 was a cohort study (LE 4). Regarding the investigated population, the majority focused on hospital nurses, although the populations of the cohort study and the German RCT were nursing professionals in different work environments. The Dutch ECR investigated other health workers in addition to nurses (Table 1).

### INTERVENTION PROGRAMS

Five different Internet-based stress management intervention programs for nurses were identified.

The program “BREATHE: Stress Management for Nurses” was developed to provide guidance and tools for managing stressors in the work environment. The program includes interactive exercises, downloadable tools, videos with the stories of real nurses, and other audiovisual content. It is organized into 7 modules, addressing changes in how to view stressors; the impact of stress on the body; stress assessment/identification of stressors; practical stress management tools, response to stressors or stressful situations; promoting effective communication skills/taking time to grieve; and aspects related to mental health (depression/anxiety).^17^

Two smartphone programs (programs A and B) for stress management were described in Imamura et al.^18^ Program A contained free choice multi-modules, and program B had fixed-sequence modules, 1 available per week. Both programs had modules based on cognitive behavioral therapy (CBT).^18^

An Internet-based CBT intervention called “Useful mental health solutions for work and everyday life” is a stress management program consisting of 6 modules embedded in a manga comic (comic of Japanese origin). The 6 modules cover different components of CBT: the transactional stress model, self-monitoring skills, behavioral activation, cognitive restructuring, relaxation, and problem solving.^19^

The “SUPPORT Coach” program, which was designed to reduce post-traumatic stress, consists of 5 sections: a) information: psychoeducation about trauma, post-traumatic stress, and professional care; b) finding support: facilitates contact with the user’s personal network and professional assistance; c) self-test: contains a list for evaluating and monitoring post-traumatic stress severity; d) calendar: allows users to schedule self-tests, exercises, and activities; and finally e) symptom management: CBT-based exercises to self-manage post-traumatic stress.^21^

“Mental Contrast with Implementation Intentions” is a mental exercise resource for stress self-regulation.^22^ The Benevolent Midwifery Project consists of 16 modules of interactive webpages with photographic demonstrations of exercises, audio files, videos, and written instructions. It is structured around the holistic modality of yoga, meditation, and mindfulness-based stress reduction techniques.^20^

### INTERVENTION APPROACHES

Although most of the studies were based on CBT approaches to stress management in the work environment,^17,18,19,21^ other approaches were also found: holistic health care, the mind-body-spirit connection, which is covered in the theory of Watson^20^ and a combination involving Mental Contrast with Implementation Intentions.^22^

### INSTRUMENTS USED TO ASSESS MENTAL HEALTH

Different measurement instruments were used to assess aspects of mental health. The Beck Depression Inventory-II was used to assess depressive symptoms, the World Health Organization’s Composite International Diagnostic Interview v 3.0^19^ was used for episodes of major depression, and the Depression Anxiety and Stress Scale was used for symptoms of depression, stress, and anxiety.^18^

Perceived stress was measured using the Perceived Self Stress scale^20^ and the Perceived Stress Questionnaire-20,^22^ while post-traumatic stress disorder was measured using the Primary Care Post-traumatic Stress Disorder Screen revised to Diagnostic and Statistical Manual of Mental Disorders, Fifth Edition criteria.^21^ The Nursing Stress Scale and the Work Limitations Questionnaire were used to assess the specific stress of nursing work.^17^ Job satisfaction was assessed with the Nurse Job Satisfaction Scale,^17^ while work engagement was assessed with the Utrecht Work Engagement Scale^22^ and the Utrecht Work Engagement Scale-Japanese version.^18,19^

Psychological stress was assessed using the Kessler Psychological Distress Scale, psychosocial aspects of work were assessed through Job Content Questionnaire, job performance was assessed with

Work Performance Questionnaire,^18,19^ and coping self-efficacy was assessed with the Coping Self-Efficacy Scale.^20^ Finally, the EuroQol-5 Dimension-5 Level questionnaire^18^ was used to assess quality of life, while physical symptoms of stress, were assessed with the Burnout Screening Scales II Inventory.^22^

## DISCUSSION

This review demonstrated the gap in the national and international literature regarding Internet-based mental health interventions for nurses. The included studies contained relevant information about the interventions, based on the hypothesis that Internet-based mental health programs for nurses would result in lower levels of depressive symptoms, lower risk of major depressive episodes,^19^ and improved psychosocial aspects of work, work engagement, and work performance^18,19^ in addition to reduced stress symptoms.^17,18,22^

However, 2 of the studies referred to trial protocols under development,^18,19^ while 3 others reported completed trials. One trial confirmed the hypothesis that “BREATHE: Stress Management for Nurses” would effectively reduce stress among nurses in American hospitals, given the significant differences between the experimental and control groups in the overall Nursing Stress Scale scores (t = −2.95; p = 0.00), as well as in 6 of the 7 subscales: death and dying (t = −2.24; p = 0.03), conflict with doctors (t = −2.11; p = 0.04), inadequate preparation ( t = −1.95; p = 0.05), conflict with other nurses (t = −4.17; p = 0.00), workload (t = −2.30; p = 0.02), and uncertainty regarding treatment (t = −2.14; p = 0.03).^17^

Another trial studied health workers, including nurses; however, a stratified analysis was not performed to assess the impact of the “SUPPORT Coach” program specifically among nurses. Nevertheless, it had relevant results regarding secondary outcomes and the intervention program, such as reduced negative cognition and increased psychological resilience to post-traumatic stress.^21^

Stress, work overload, long working hours, and precarious working conditions make the workplace a potential contributor to mental illness. It has been observed that nurses are exposed to a stressful work environment, work overload, and constant tension, in addition to precarious environmental conditions,^2^ and are often affected by symptoms of depression.^2,5^

Kuribayashi et al.^19^ reported a study protocol on the effectiveness of an Internet-based program to reduce depressive symptoms and prevent major depression in hospital nurses in Japan. The CBT program “Useful mental health solutions for work and everyday life” was designed in the form of a manga comic, which indicates the importance of cultural context as a didactic strategy. This program, which was based on the CBT approach to stress management and depressive symptoms^19^ and had effectively improved these symptoms in a previous trial, was administered to healthy workers.^14^

From a similar perspective, the protocol by Imamura et al.^18^ involves an Internet-based intervention to reduce depressive symptoms and anxiety among nurses in Vietnam. The intervention consists of 2 multi-module stress management smartphone apps. One addresses behavioral activation, cognitive restructuring, problem solving, assertiveness, self-compassion, and job crafting. The other is related to the transactional model of stress and coping, self-case formulation based on the cognitive behavioral model, behavioral activation skills, cognitive restructuring skills, problem-solving skills and relaxation skills.^18^ Another systematic review and meta-analysis study of general workers also reported on the use of smartphone apps, as well as other Internet-based interventions. The interventions resulted in positive effects and were based on stress management strategies, mindfulness, and CBT.^23^

The importance of CBT in mental health interventions for nurses is evident, given its effectiveness for stress management in other workers.^13^ However, therapy based on the holistic health care model also reduced stress in obstetric nurses.^20^ Guided by the theoretical model of Watson,^24^ it involves the following steps: humanistic-altruistic values; faith-hope; sensitivity to oneself and others; human care relationships that engender trust; the expression of positive and negative feelings; creative problem-solving processes; transpersonal teaching-learning; a supportive, protective, and corrective mental, physical, social, and spiritual environment; human assistance needs; and existential-phenomenological-spiritual forces.^20^

Unlike the aforementioned approaches, the intervention in Gollwitzer et al.^22^ used the logic of decision autonomy from the perspective of self-regulation of stress, which is based on the Mental Contrast with Implementation Intentions strategy. In this approach, the nurses themselves detected their desire to reduce stress and were motivated to identify what prevented them from reducing it. After identifying the obstacles, they outline what they wanted to do to overcome them. This study involved no specific program, but rather a website to apply of measurement instruments and mental exercise instructions in the form of commands and questions.^22^

In this panorama of mental health interventions for nurses based on information and communication technology, the Brazilian Federal Nursing Commission developed a Mental Health in Nursing Care project during the COVID-19 pandemic in response to wear and tear on the mental health of frontline nurses. Unlike the studies identified here, this live chat program with nurses who are mental health experts involves empathetic listening based on humanist theory to assist nursing professionals 24 hours a day.^3^

Regarding the measurement instruments of the included studies, only Hersch et al.^17^ assessed stress from the specific perspective of nursing work, using the Nursing Stress Scale. This scale was designed to assess sources of stress in the following 7 subscales: death and dying, conflict with doctors, inadequate preparation, lack of support, conflict with other nurses, workload, and uncertainty about treatment. The Nursing Stress Scale provides a total stress score, as well as scores for each subscale to measure how often the top 7 sources of stress are experienced by nurses.^25^ In the other studies, the instruments assessed work stressors, such as the Job Content Questionnaire and the Work Limitations Questionnaire, although they did not consider the particularities of nursing work.

These instruments, as well as others that assessed the studies’ primary and secondary outcomes, were applied at baseline and after 3 weeks,^22^ 3 months,^17^ 3 and 7 months^18^ and 3 and 6 months.^19^ The intervention periods varied: 4 weeks,^20^ 9 weeks,^19^ 10 weeks,^18^ and 12 weeks.^17^ Nurses were recruited through information published in the hospital’s intranet, leaflets distributed in the units,^17^ and through direct email invitation.^17,18,19,22^

In general, the interventions in this review had a positive effect on the mental health of nurses. However, some considerations should be highlighted about bias, such as some studies’ use of financial incentives to attract participants. Such a strategy can create bias among the participants, and may lead to overestimates of illness. Another limiting factor identified in most of the studies was the online application of measurement instruments, which can result in incomplete data. Furthermore, stress management apps may not be based on scientific evidence. For example, a study analyzing 902 apps in the iOS App Store found that most met the criteria of usability and transparency and 31% were affiliated with non-profit research institutions, but only 3.54% were evidence-based.^26^

It should also be pointed out that double blinding was not possible in the included clinical trials, given that the interventions were developed from a psychotherapeutic perspective. However, given the importance of methodological rigor, most of the trials in this review described the randomization process in detail, which is essential for reducing the risk of selection bias and increasing the reliability of the results.

### STUDY LIMITATIONS

This review also has some limitations that should be considered: a) limiting the number of languages may have excluded some relevant articles; b) other relevant studies may have been indexed in other databases we did not search, or could have been published in event annals, as books, or as theses/dissertations/monographs; c) studies unavailable electronically may not have been identified; and d) our combinations of search terms may not have effectively covered all relevant publications.

### CONTRIBUTIONS TO THE AREA

The occupational stress, work overload, and emotional exhaustion to which nursing professionals are exposed has intensified during the pandemic, resulting in greater vulnerability to mental illness, such as depression, anxiety,^11,12^ and sleep disorders.^11^ Thus, the need to care for their mental health is increasing. This review highlighted the necessary parameters for strengthening studies on the health of health care workers, giving visibility to the issue of mental illness among nurses and, above all, highlighting how Internet-based interventions are a potential strategy for mental health promotion at the primary health care level.

## CONCLUSIONS

This review of Internet-based mental health interventions for nurses found that they use psychotherapeutic methods and mental health education as a way of preventing and controlling stress, anxiety, and depression. However, the few studies found on the subject reveal a gap in the literature regarding other approaches to Internet-based mental health interventions, such as art, culture, and entertainment as prevention and rehabilitation strategies. Thus, new studies should be conducted on the effectiveness of Internet-based mental health interventions in nurses, contributing new preventive and therapeutic alternatives for psychiatric disorders.
